# Quantitative Analysis of Bradykinesia and Rigidity in Parkinson’s Disease

**DOI:** 10.3389/fneur.2018.00121

**Published:** 2018-03-06

**Authors:** Lazzaro di Biase, Susanna Summa, Jacopo Tosi, Fabrizio Taffoni, Massimo Marano, Angelo Cascio Rizzo, Fabrizio Vecchio, Domenico Formica, Vincenzo Di Lazzaro, Giovanni Di Pino, Mario Tombini

**Affiliations:** ^1^Neurology Unit, Campus Bio-Medico University of Rome, Rome, Italy; ^2^NeXT: Neurophysiology and Neuroengineering of Human-Technology Interaction Research Unit, Università Campus Bio-Medico, Rome, Italy; ^3^Nuffield Department of Clinical Neurosciences, University of Oxford, John Radcliffe Hospital, Oxford, United Kingdom; ^4^Unit of Biomedical Robotics and Biomicrosystems, School of Engineering, Campus Bio-Medico University of Rome, Rome, Italy; ^5^Brain Connectivity Laboratory, IRCCS San Raffaele Pisana, Rome, Italy; ^6^IRCCS San Raffaele Pisana, Rome, Italy

**Keywords:** parkinson’s disease, wearable sensors, quantitative analysis, kinematic analysis, parkinson’s disease diagnosis

## Abstract

**Background:**

In the last decades, several studies showed that wearable sensors, used for assessing Parkinson’s disease (PD) motor symptoms and recording their fluctuations, could provide a quantitative and reliable tool for patient’s motor performance monitoring.

**Objective:**

The aim of this study is to make a step forward the capability of quantitatively describing PD motor symptoms. The specific aims are: identify the most sensible place where to locate sensors to monitor PD bradykinesia and rigidity, and identify objective indexes able to discriminate PD OFF/ON motor status, and PD patients from healthy subjects (HSs).

**Methods:**

Fourteen PD patients (H&Y stage 1–2.5), and 13 age-matched HSs, were enrolled. Five magneto-inertial wearable sensors, placed on index finger, thumb, metacarpus, wrist, and arm, were used as motion tracking systems. Sensors were placed on the most affected arm of PD patients, and on dominant hand of HS. Three UPDRS part III tasks were evaluated: rigidity (task 22), finger tapping (task 23), and prono-supination movements of the hands (task 25). A movement disorders expert rated the three tasks according to the UPDRS part III scoring system. In order to describe each task, different kinematic indexes from sensors were extracted and analyzed.

**Results:**

Four kinematic indexes were extracted: fatigability; total time; total power; smoothness. The last three well-described PD OFF/ON motor status, during finger-tapping task, with an index finger sensor. During prono-supination task, wrist sensor was able to differentiate PD OFF/ON motor condition. Smoothness index, used as a rigidity descriptor, provided a good discrimination of the PD OFF/ON motor status. Total power index, showed the best accuracy for PD vs healthy discrimination, with any sensor location among index finger, thumb, metacarpus, and wrist.

**Conclusion:**

The present study shows that, in order to better describe the kinematic features of Parkinsonian movements, wearable sensors should be placed on a distal location on upper limb, on index finger or wrist. The proposed indexes demonstrated a good correlation with clinical scores, thus providing a quantitative tool for research purposes in future studies in this field.

## Introduction

Parkinsons’ disease (PD) diagnosis, staging, and clinical grading, to date, rely on clinical evaluation. Motor symptoms, such as bradykinesia, resting tremor, and rigidity, are hallmarks for the assessment and evaluation of the disease. With the disease progression, daily patients motor status starts to fluctuate between ON and OFF, i.e., to a status when the motor symptoms are adequately controlled by therapy, to a status when motor impairments are more evident. In order to control these motor symptoms changes, with a personalized and fine-tuned therapy, a precise clinical rating is needed, thus requiring periodic clinical visits.

Moreover, clinical diaries can help to evaluate the global motor performance; however, they are affected by poor objectiveness (1), and low compliance. The most objective and standardized clinical evaluation available, is based on semiquantitative scoring system, by means of clinical rating scales like UPDRS (2) or the more recent MDS-UPDRS (3). To date, using current diagnostic criteria (4), even for a neurologist expert of movement disorders, the error rate in the diagnostic accuracy can be estimated around 20% (5). The most relevant problems related to PD clinical evaluation are that: it is a time-consuming activity; it is not objective; to make it reliable a movement disorders expert is needed; it is not remotely administrable. All these issues lead to high direct and indirect cost for the health system and for the patients.

In the last years, the spread of low cost and non-invasive technologies for motion analysis, such as magneto-inertial wearable devices, brings to new methods for the assessment of pathologies characterized by motor dysfunction. Modern technologies like wearable sensors can provide a not invasive, accurate, rapid, remote, low cost, operator independent, objective, and scalable system. The idea to monitor pathological motion deficits using wearable sensors dates back to 1950s (6), and its application for PD patients started in 1990s (7). Although their clinical use is not so common yet, wearable motion sensors are largely used with the purpose of measuring movement and physiological signals. However, further work is needed to validate these systems and bring them to the everyday clinical practice. The cardinal motor symptoms of PD patients are bradykinesia, resting tremor, and rigidity (4). Bradykinesia is considered the most important and representative of the motor symptoms, and is defined as slowness in the initiation of voluntary movement with progressive reduction in speed and amplitude of repetitive actions (4). Following the definition of bradykinesia, the fatigability of speed and amplitude, is a core feature; however, this is not a simple feature to catch with clinical evaluation, but it can be detected through an instrumented quantitative evaluation. The most studied cardinal symptom, by means of sensors, is the tremor, and in the last years there are several studies that have explored the characteristics of PD tremor (8, 9) in order to allow differential diagnosis with other tremor syndromes (10), or simply to monitor fluctuations of this symptom. Finally, rigidity is the most challenging motor symptom, to measure in an objective way, and only few studies have explored the accuracy of instrumental evaluation of rigidity with different devices (9).

To ensure proper monitoring of PD motor symptoms, a wearable system must be able to discriminate healthy subjects (HSs) from PD patients as well as to differentiate the ON from the OFF motor status in PD patients. In literature, among studies focused on the use of wearable sensors in PD, there is a lot of variability about the body distribution of sensors and about the specific indexes used to sense cardinal motor symptoms.

The aim of this study is to make a step forward the capability of quantitatively describing PD motor symptoms. In particular, the aims of the present study are: identify the most sensible place where to locate sensors to monitor PD bradykinesia and rigidity, and identify objective indexes able to discriminate PD patients from HS, and able to differentiate in PD patients ON from OFF motor status.

## Materials and Methods

### Subjects

Fourteen PD patients (Table 1) (8 male, age: 67 ± 6 years) were enrolled in the study to evaluate bradykinesia and rigidity. Inclusion criteria for PD patient group were: a possible-probable diagnosis of PD according to UK PD Brain Bank criteria (4) and an Hoehn and Yahr (11) stage between 1 and 2.5. Exclusion criteria for PD patient was Hoehn and Yahr stage higher than 2.5; and for both PD and HSs group, another exclusion criteria was limitation of the physiological joints range of motion caused by other pathologies.

**Table 1:** Parkinsons’ disease patients characteristics.

ID	Disease duration (years)	Gender	Dominant hand	Most affected side	LEDD (mg)	UPDRS part III score (OFF condition)
S1	10	M	Right	Right	750	45
S2	5	M	Right	Left	750	29
S3	9	F	Right	Left	950	29
S4	7	F	Right	Right	660	36
S5	21	F	Right	Left	660	29
S6	10	M	Right	Right	925	65
S7	4	F	Right	Left	550	23
S8	NA	M	Left	Left	NA	49
S9	7	M	Right	Right	700	20
S10	2	F	Right	Right	300	25
S11	7	F	Right	Left	1,150	29
S12	6	M	Right	Left	600	25
S13	10	M	Right	Left	700	27
S14	7	M	Right	Right	670	43

*M, male; F, female; LEDD, levodopa equivalent daily dose; NA, not available; S1-14, subject number*.

Thirteen age-matched HSs composed the control group (seven males, age: 69 ± 19 years).

The research was carried out in accordance with the Declaration of Helsinki. All patients and control subjects gave informed consent and the study was approved by local research ethics committee.

For PD group patients, kinematic analysis was performed for the most affected arm. For the HS group, the kinematic analysis was performed on dominant arm, identified with the Oldfield test (12).

Parkinsons’ disease subjects were analyzed twice: after 12 h withdrawal of any medications (OFF motor condition) and after 1 h from administration of 150% of patient’s l-dopa morning dose (ON motor condition).

### Motor Tasks

Subjects were sitting in a chair and were asked to perform three motor tasks from the UPDRS part III. Rigidity (UPDRS task 22) was tested, without an activation maneuver, on slow passive movement of elbow joints, with the patient in a relaxed position. During this task, the examiner, holding against gravity the arm, moved the forearm for 10 times for each side. Bradykinesia was evaluated performing two task: finger tapping (UPDRS task 23) and prono-supination movement of the hands (UPDRS task 25). During the finger-tapping task, subjects were asked to tap the index finger on the thumb 15 times as quickly and as big as possible; each side was evaluated separately. During the prono-supination task, subjects were asked to extend the arm out in front of them with the palms down; then to turn the palm up and down alternately 15 times as fast and as fully as possible.

A movement disorders expert rated the three tasks according to the UPDRS part III scoring system.

### Data Acquisition

**Figure 1:**
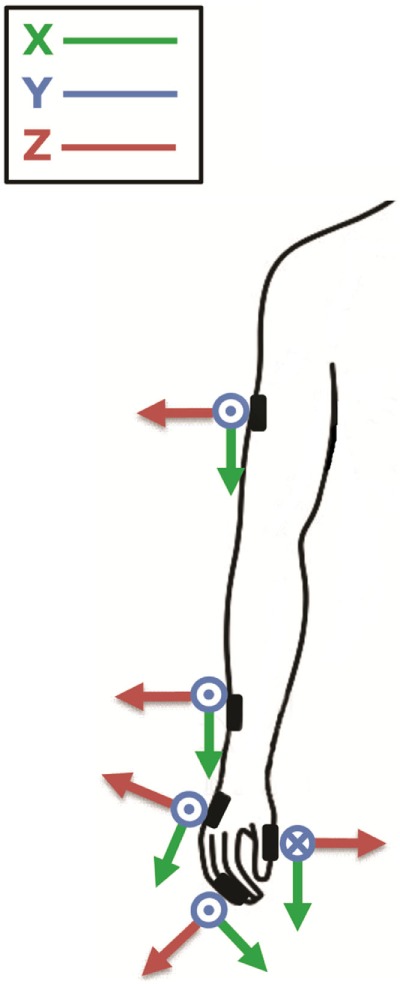
The figure shows where sensors were placed; second phalanx of the index finger, thumb, metacarpus, wrist, and arm; and their orientation around the three axes (*x, y, z*). Modified from Ref. (13).

In order to identify the most informative parameters to describe PD motor symptoms, motor tasks were recorded using five magneto-inertial units (M-IMU, device OPAL, APDM, Inc., Portland, OR, USA) and a camera GoPro Hero4 (GoPro, San Mateo, CA, USA). The data acquisition software MotionStudio (APDM Inc., Portland, OR, USA) allowed the camera to be synchronized with the sensors. The experiments were performed positioning sensors on the following anatomical landmark: second phalanx of the index finger (with the sensor *X*-axis in line with the same bone), distal phalanx of thumb (with the sensor *X*-axis in line with the same bone), metacarpus (fixed at medium point of third metacarpal bone, on the dorsal metacarpus, with the sensor *X*-axis in line with the third metacarpal bone), wrist (fixed at the medium point between radius and ulna bones, on the most distal dorsal part of radius and ulna bones, with the sensor *X*-axis in line with the radius bone), and arm (fixed at medium point between the greater tubercle of the humerus and its lateral epicondyle, with the sensor *X*-axis in line with the homerus) (Figure 1).

### Data Analysis

According to the literature (14, 15), raw data were first high-pass filtered with a cut-off frequency of 1 Hz, to remove the effect of gross changes in the orientation of body segments. Moreover, the frequency component of interest for estimating each symptom can be isolated; specifically for tremor a bandpass filter with bandwidth 4–8 Hz was used, while for bradykinesia data were band pass between 1–4 Hz.

In the task used to assess bradykinesia severity, we defined a movement cycle as the set of submovements needed to complete the task for one repetition. For instance, a finger-tapping cycle consists of starting with the hand opened, closing, and then opening the fingers to the initial position for one time. We estimated the movement time as the beginning and end of cycles, identified from the speed profile with a threshold of 10% of the peak value of each cycle. In addition, we calculated the total time needed to complete the full task (Eq. 1) as:

(1) $$t_{\text{TOT}}=t_{b}-\;t_{a}$$

Total time: it is difference between the end time (*t_b_*) of the last cycle and the begin of the first cycle (*t_a_*).

From gyroscope signals in the time domain, we estimated the peak-to-peak values of angular velocity for all three axes. In order to capture the progressive reduction in speed amplitude, we perform a first-order regression between these peak-to-peak values and the progressive number of cycles. We defined the fatigability index as the slope of the fitted linear equation (Eq. 2):

(2) y=mx + q

Fatigability index: it is the slope (*m*) of the linear equation fitted with the peak-to-peak values of angular velocities extracted from the gyroscope signals.

Of note, for the fatigability index we consider for each task only the gyroscope axis most relevant for that specific task.

As regards the frequency domain, we extracted the total power (the integral of the power spectrum) from the power spectrum density (PSD) of angular velocity as suggested by Kim et al. (16). In fact, one of the results of Fourier analysis is the Parseval’s theorem, which states that the area under the energy spectral density curve is equal to the area under the square of the magnitude of the signal, i.e., the total energy. A similar result holds for power. The total power is expected to represent the overall intensity of movement.

**Figure 2:**
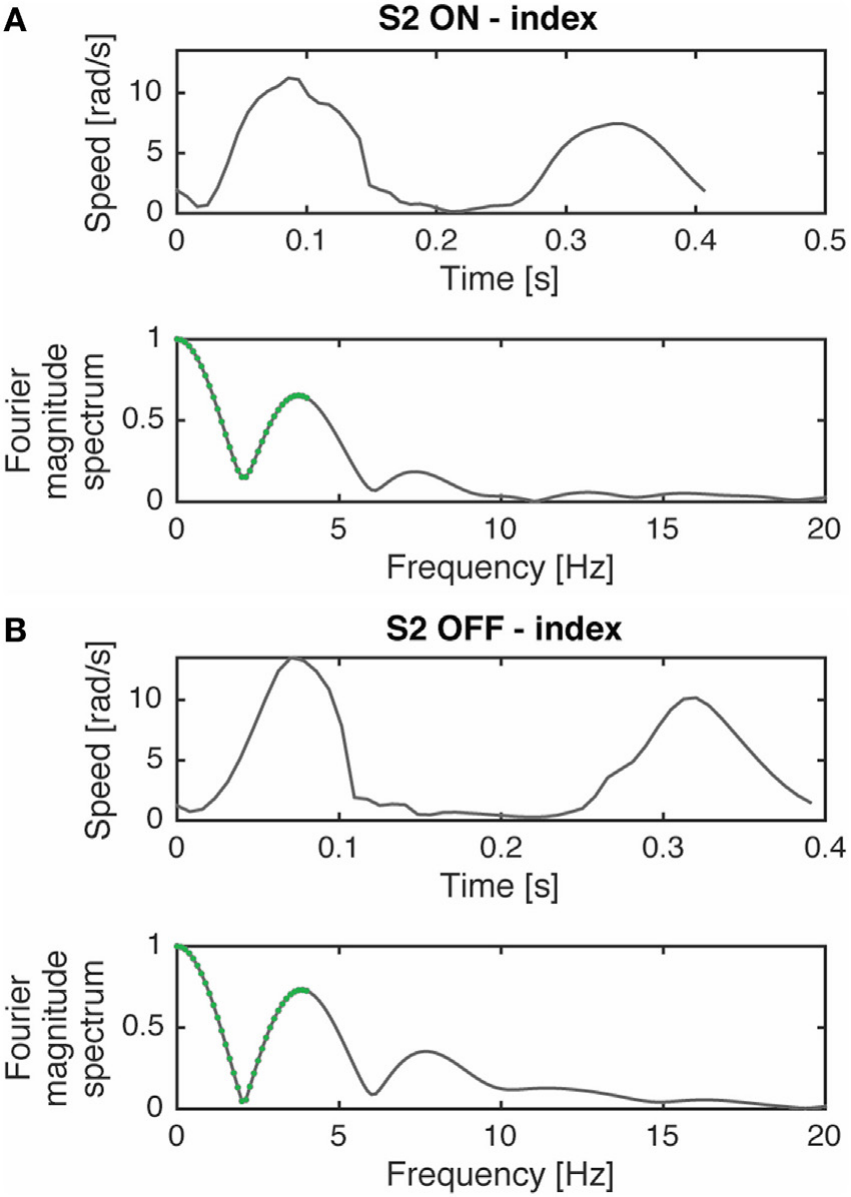
Movement speed profile, during a prono-supination task, of a typical subject (S2) in ON **(A)** and OFF **(B)** phases and their respective Fourier magnitude spectrum. The segments used for computing spectral arch length are highlighted in green. The complexity of the Fourier magnitude spectrum changes with the submovement characteristics variations (i.e., inter-submovement interval) of the movement speed profile, as already shown by Ref. (17). Modified from Ref. (13).

We also introduced a smoothness parameter as a bradykinesia descriptor. According to previous studies (17, 18), we measured smoothness using the spectral arch length (SAL) of movement speed profile as an appropriate index of movement fluidity (Figure 2). We decided to look at this type of smoothness measure because Balasubramanian et al. (17) showed that the SAL can account for the change in the number of submovements and the inter-submovement interval, which are movement features influenced by bradykinesia. As explained by the authors (17, 18), to compute smoothness it was not necessary to filter data because of the inherent low-pass filtering action performed. Specifically, we compute the SAL within the frequency range 0–4 Hz of the speed profile in each movement cycle and in each single movement.

Spectral arch length estimates smoothness by calculating the arc length of the magnitude of the Fourier Spectrum of a given speed profile *v*(*t*), within a frequency range (0 − ω*_c_*) (Eq. 3). Definition of SAL as introduced by Ref. (17, 18):

(3) $$\text{SAL }\underline{\underline{\Delta }}\;-{\int_{0}^{\omega_{c}}\left[{\left(\frac{1}{\omega_{c}}\right)}^{2}+{\left(\frac{\text{d}\hat{V}(\omega )}{\text{d}\omega }\right)}^{2}\right]}^{\frac{1}{2}}d\omega ;\;\hat{V}(\omega )=\frac{V(\omega )}{V(0)},$$

where *V*(ω) is the magnitude of the Fourier spectrum of *v*(*t*), $\hat{V}(\omega )$ is the spectrum magnitude normalized with respect to the magnitude at zero frequency *V*(0), and ω*_c_* is adaptively selected based on the following equation:

(4) $$\omega_{c}\;\underline{\underline{\Delta }}\;\min\left\{\omega_{c}^{\text{max}},\text{ min}\left\{\omega ,\hat{V}(r)<\bar{V}\forall r>\omega \right\}\right\}$$

Definition of the frequency value ω*_c_* for the calculus of the SAL in Eq. 3.

Equation 4 defines ω*_c_* as the minimum value between: (i) an upper bound limit for this parameter $\omega_{c}^{\text{max}}$, which has been set in our analysis to 4 Hz; (ii) the value of frequency above which the normalized spectrum magnitude is always lower than a given threshold $\bar{V}$, set in our analysis to 10%.

The definition of the SAL modified with the adaptive parameter ω*_c_* is referred in literature as the SPARC index (18).

Finally, we investigated the relationship between this index and the elbow joint rigidity and we tested the smoothness index as rigidity descriptor. In PD, the classic cogwheel rigidity causes a fragmentation and decomposition of the passive movement, leading to less smooth than normal passive movements. We estimated the beginning and the end of movement looking at the speed profile with a threshold of 10%. Then we computed the averaged SAL of movement speed profile for each movement and looked at differences between OFF and ON motor statuses. Specifically, we computed the SAL within the frequency range 0–20 Hz of the speed profile in each movement cycle.

### Statistical Analysis

First, we looked for normality of distributions with the Shapiro–Wilk test, because of the number of participants in each group (19).

As mentioned previously, we are investigating those indexes able to differentiate the OFF motor status from the ON one in PD patients; moreover, we want to identify the most sensible place where to locate sensors. Therefore, for each indicator we conducted two-way repeated measures ANOVA with state (ON vs OFF) and sensor locations—5 levels in bradykinesia tasks (arm, index, metacarpus, thumb, and wrist) and two levels in rigidity task (left wrist and right wrist)—as within-subject factor. We additionally assessed the most sensible place where to locate sensors while discriminating the state factor. That is, the multiple comparisons of the interactive effect state × sensor location.

For OFF vs HS discrimination, data were analyzed using a mixed-design ANOVA with a within-subjects factor sensor location and a between-subject factor the group (OFF or Healthy). For ON vs HS discrimination, we ran a mixed-design ANOVA with a within-subjects factor sensor location and a between-subject factor the group (ON or Healthy). We additionally assessed the most sensible place where to locate sensors while discriminating the group factor. That is, the multiple comparisons of the interactive effect group × sensor location.

Sensor location in bradykinesia task (arm, index, metacarpus, thumb, and wrist).

In order to identify the most sensible place where to locate sensors, to monitor PD bradykinesia and rigidity, while excluding false-positive results under multiple testing, we applied Bonferroni correction and *p*-values were compared against α/(number of comparison) instead of α = 0.05.

For total time index, we used a paired-sample *t*-test, to test its capability to differentiate the ON/OFF motor status. Similarly, we used an independent sample *t*-test to see if total time index was capable to discriminate HS from the OFF or from the ON condition.

We also looked at the correlation of each indicator in the OFF and the ON condition, with the UPDRS part III scale. Correlation was reported as *R*-squared values.

For data analysis was used Matlab (Mathworks, Natick MA, USA).

## Results

### Clinical Rating

The results of clinical rating with UPDRS part III scale, for each task are shown in Tables 2 and 3, respectively, for bradykinesia and rigidity task.

**Table 2:** Bradykinesia task clinical rating.

Subjects	UPDRS part III score			
	Task 23, finger tapping		Task 25, arm prono-supination	
	OFF	ON	OFF	ON
S1	3	3	2	2
S2	2	1	1	1
S3	1	0	1	0
S4	2	1	2	1
S5	1	1	1	1
S6	3	2	4	4
S7	1	0	1	0
S8	3	2	2	2
S9	2	1	1	1
S10	1	1	1	1
S11	2	1	1	0
S12	2	1	2	1
S13	1	1	1	1
S14	3	2	1	1

**Table 3:** Rigidity task clinical rating.

Subjects	UPDRS part III score, Task 22			
	Right arm rigidity		Left arm rigidity	
	OFF	ON	OFF	ON
S1	2	2	2	2
S2	2	1	2	1
S3	2	2	2	2
S4	2	1	1	1
S5	2	1	2	2
S6	2	2	2	2
S7	1	0	1	1
S8	2	2	3	2
S9	0	0	0	0
S10	2	1	2	1
S11	1	1	2	1
S12	1	1	2	2
S13	1	1	2	1
S14	2	2	1	1

### Kinematic Index

Parkinsons’ disease patients S13 and S14 were excluded from the kinematic analysis due to artifacts into accelerometric signal.

#### Movement Time

**Figure 3:**
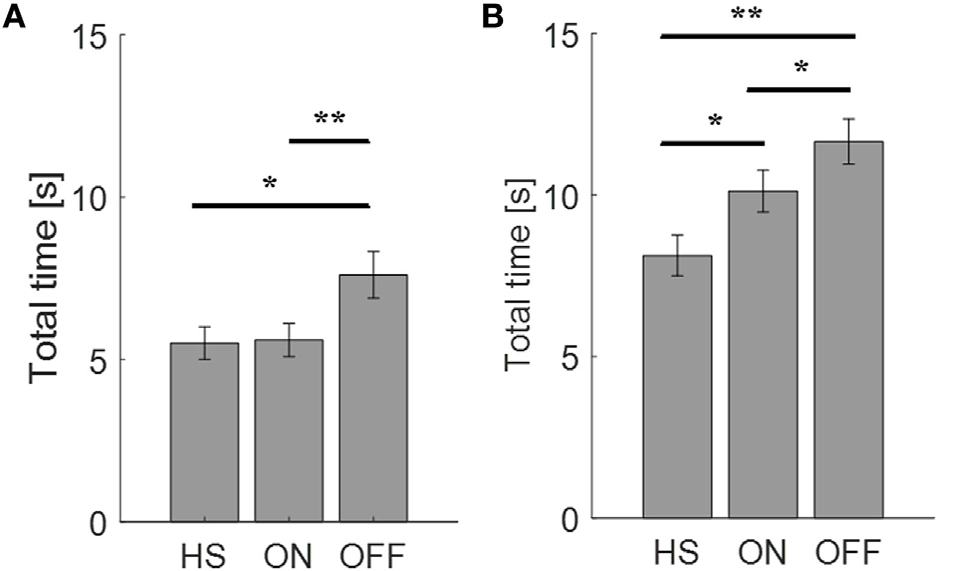
Total time needed (seconds) to complete the finger-tapping **(A)** and the arm prono-supination tasks **(B)**. Values are averaged for each group. Bars denote the SE. *p < 0.05 (*t*-test); **p < 0.01 (*t*-test).

First of all, we looked at the total time needed to complete the finger-tapping and the arm prono-supination tasks. In the OFF condition, PD subjects needed more time to complete the task than during the ON condition. Considering the arm prono-supination task, statistical analysis showed that the total time is able to discriminate the OFF vs ON motor condition (*p* = 0.01) and also to differentiate the HS group from PD patients in OFF and ON conditions (OFF *p* = 0.001; ON *p* = 0.04) (Figure 3B). For the finger-tapping task, the total time is able to discriminate the OFF vs ON motor condition (*p* = 0.001) and also to differentiate the HS group from PD patients in OFF condition (*p* = 0.02) (Figure 3A). Good correlations were found in the finger-tapping task, between total time and UPDRS item 23 score, for both motor conditions (OFF *R*^2^ = 0.34; ON *R*^2^ = 0.74). No correlation was found between total time and UPDRS item 25 score in arm prono-supination task.

#### Peak-to-Peak Velocity and Fatigability

**Figure 4:**
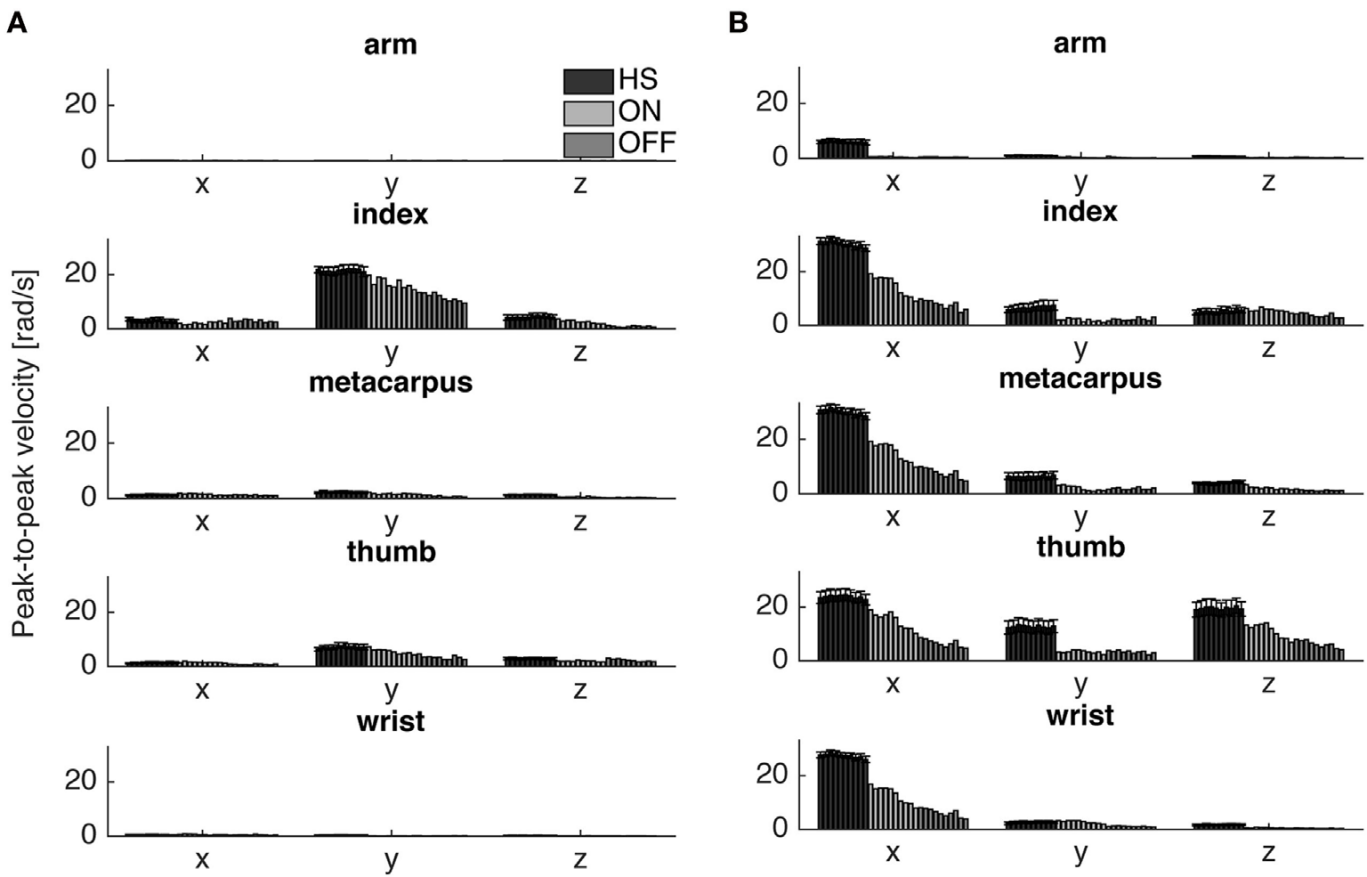
Peak-to-peak velocity of all cycles of a typical Parkinson’s disease subject (S6) in comparison with the averaged values of healthy subject (HS) group, for the finger-tapping **(A)** and the arm prono-supination tasks **(B)** for each gyroscope channel. Values HS group values are averaged and bars denote the SE.

To catch the whole kinematic information related to the task performed is important to consider where to place the sensor and which orientation axis use for analysis. For finger-tapping task, the *y*-axis was chosen for the analysis, instead for the prono-supination task the *x*-axis was the most informative (Figure 4). Therefore, we looked at the fatigability on these two axes depending on the task (Tables 4 and 5).

**Table 4:** Finger-tapping task kinematic results.

	Kinematic index		
	Fatigability	Total power	Fatigability
State (PD ON vs PD OFF)		**	**
Sensor location		**	**
State (ON/OFF) × sensor location interaction		**	
State (PD OFF vs HS)		**	**
Sensor location		**	**
State (OFF/HS) × sensor location interaction		**	
State (PD ON vs HS)			
Sensor location		**	**
State (ON/HS) ×sensor location interaction		*	

*p < 0.05 (ANOVA); **p < 0.01 (ANOVA).

*PD ON, Parkinson’s disease patients in ON motor status; PD OFF, Parkinson’s disease patients in OFF motor status; HSs, healthy subjects*.

**Figure 5:**
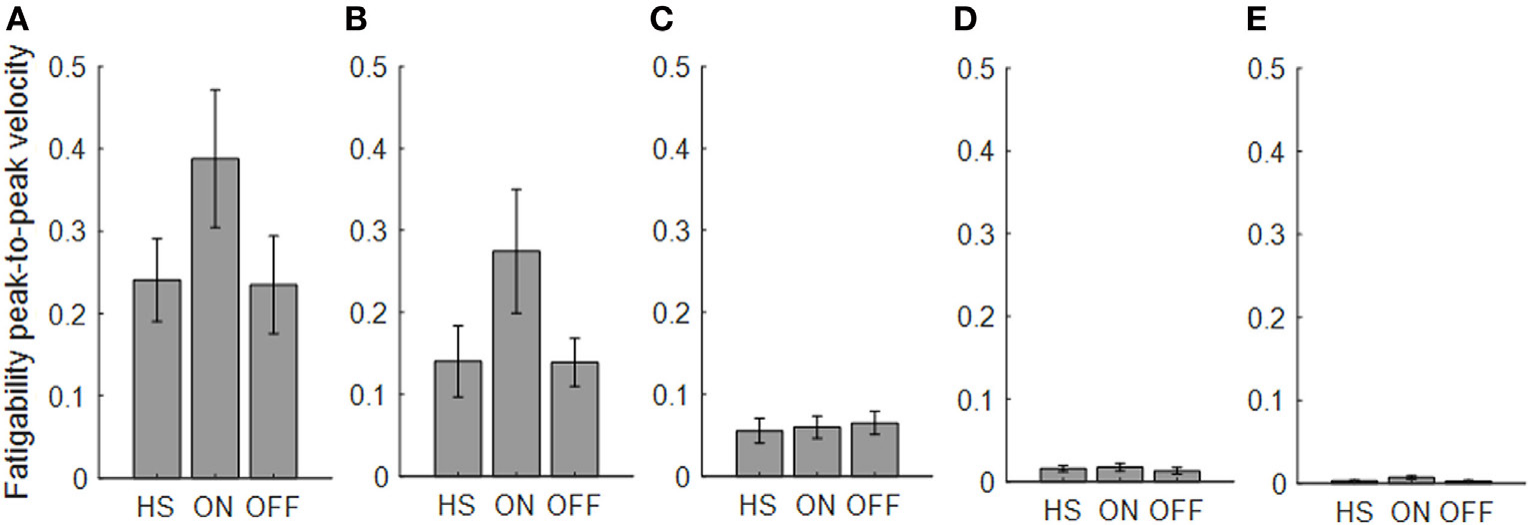
Fatigability index computed from the finger-tapping task. Each panel shows the averaged values for each group OFF, ON, and healthy subject (HS) for index finger **(A)**, thumb **(B)**, metacarpus **(C)**, wrist **(D)**, and arm **(E)**. Bars denote the SE.

**Figure 6:**
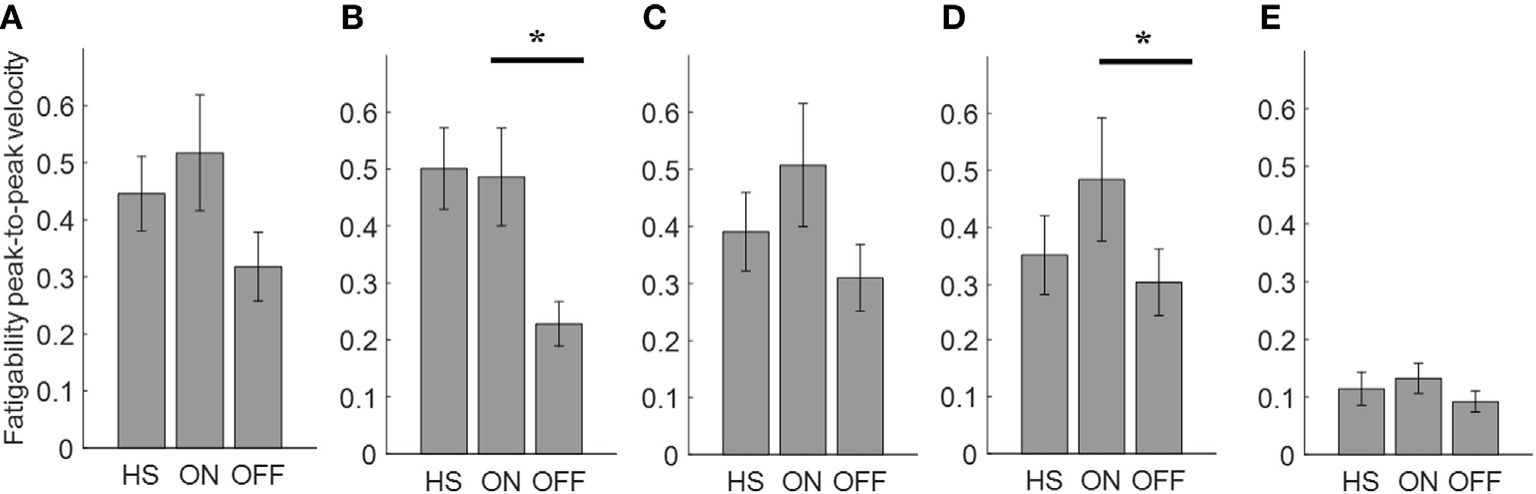
Fatigability index computed from the arm prono-supination task. Each panel shows the averaged values for each group OFF, ON, and healthy subject (HS) for index finger **(A)**, thumb **(B)**, metacarpus **(C)**, wrist **(D)**, and arm **(E)**. Bars denote the SE. Bonferroni correction *p < 0.01.

The fatigability index assessed in the finger-tapping and arm prono-supination tasks are shown, respectively, in Figures 5 and 6. As mentioned before, the fatigability represents the progressive reduction in speed of the movement, see Figure 4.

Analyzing the finger-tapping task, the ANOVA analysis showed no significant main effect of the state in none among the three state comparison, ON/OFF state, OFF/HS, and ON/HS on fatigability index (Figure 5).

Analyzing the arm prono-supination task, there was a significant main effect of ON/OFF state [*F*(1) = 9.899; *p* = 0.009] and of sensor location [*F*(4) = 8.548; *p* < 0.001] on fatigability index. There was a significant interaction between ON/OFF state and sensor location on fatigability index [*F*(4) = 3.957; *p* = 0.008].

The *post hoc* test, showed the following sensors significances, thumb *p* = 0.003; wrist *p* = 0.006.

There was no significant effect of OFF/healthy state on fatigability index, there was a significant main effect of sensor location on this index [*F*(4; 92) = 3.556; *p* = 0.01] and there was no interaction between OFF/healthy state and sensor location on this index.

In addition, there was a significant main effect of ON/healthy state [*F*(1; 23) = 5.76; *p* = 0.025] and of sensor location [*F*(4; 92) = 6.509; *p* < 0.001] on this index. There was no interaction between ON/healthy state and sensor location on fatigability index. *Post hoc* analysis showed no statistically significant differences among sensors.

We found a good correlation between the UPDRS item 23 score and fatigability measured in the finger-tapping task but only in the OFF condition for the index finger (*R*^2^ = 0.49). No correlation was found between the fatigability and UPDRS item 25 score in arm prono-supination task.

#### Total Power

**Figure 7:**
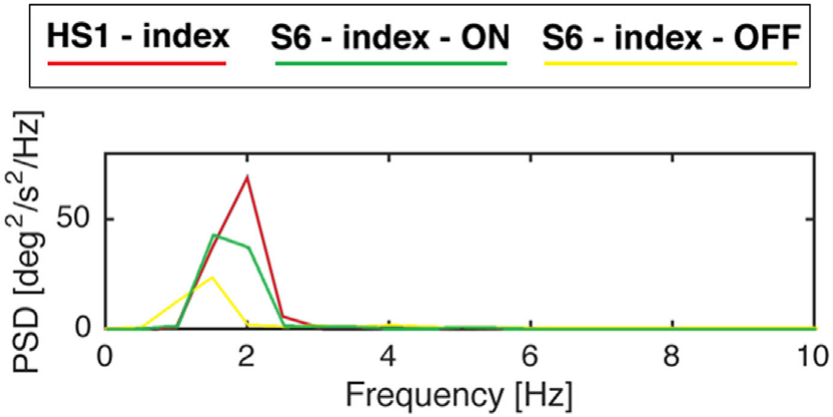
Power spectral density of gyroscope signal of subjects S6 from the Parkinson’s disease group and S1 from the healthy subject (HS) group while performing finger-tapping task. Modified from Ref. (13).

Power spectral density in PD subject in ON phase increases in amplitude compared with the one in OFF phase (Figure 7).

**Figure 8:**
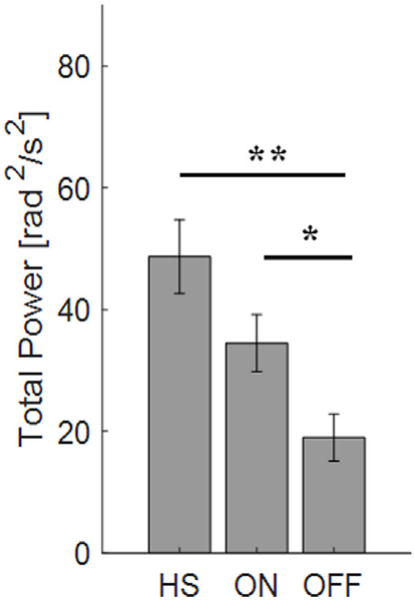
Total power index computed from the finger-tapping task, with sensor on index finger. Values are averaged for each group OFF, ON, and healthy subject (HS). Bars denote the SE. Bonferroni correction *p < 0.01; **p < 0.001.

For the finger-tapping task (Table 4), there was a significant main effect of ON/OFF state [*F*(1) = 14.047; *p* = 0.003] and of sensor location [*F*(4) = 47.709; *p* < 0.001] on total power index, with a significant interaction between ON/OFF state, and sensor location on this index [*F*(4) = 16.786; *p* < 0.001] (Table 4). *Post hoc* analysis showed the following sensors location significances, index finger *p* = 0.001 (Figure 8).

ANOVA analysis showed a significant main effect of OFF/healthy state [*F*(1; 23) = 16.247; *p* < 0.001] and of sensor location [*F*(4; 92) = 82.576; *p* < 0.001] on total power index, with a significant interaction between OFF/healthy state and sensor location on this index [*F*(4) = 15.946; *p* < 0.001]. *Post hoc* analysis showed the following sensors location significances, index finger *p* < 0.001 (Figure 8).

There was no significant effect of ON/healthy state on total power index, during the finger-tapping task.

In this task, the correlation between total power and the UPDRS item 23 score in OFF motor condition is good for the index finger (*R*^2^ = 0.57) and the thumb (*R*^2^ = 0.47).

**Figure 9:**
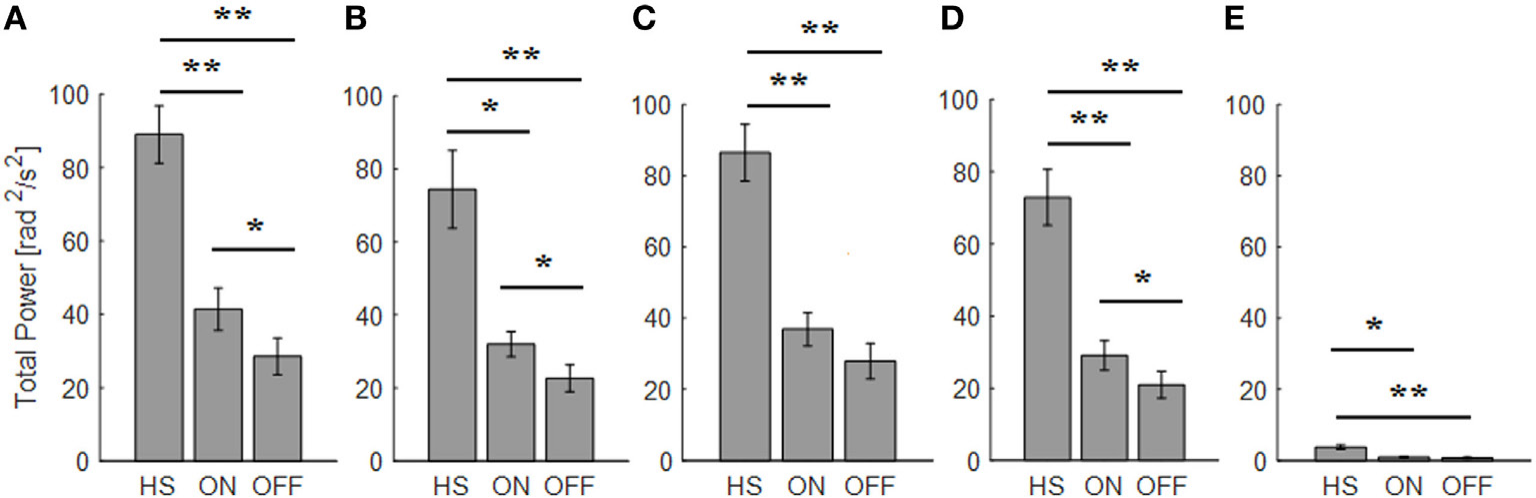
Total power index computed from the arm prono-supination task. Each panel shows the averaged values for each group OFF, ON, and healthy subject (HS) for index finger **(A)**, thumb **(B)**, metacarpus **(C)**, wrist **(D)**, and arm **(E)**. Bars denote the SE. Bonferroni correction *p < 0.01; **p < 0.001.

If we use the arm prono-supination to assess bradykinesia (Figure 9), it is possible to discriminate OFF vs ON motor status, OFF vs HS, and ON vs HS group (Table 5).

**Table 5:** Prono-supination task kinematic results.

	Kinematic index		
	Fatigability	Total power	Smoothness
State (PD ON vs PD OFF)	**	**	**
Sensor location	**	**	**
State (ON/OFF) × sensor location interaction	**	**	
State (PD OFF vs HS)		**	**
Sensor location	**	**	**
State (OFF/HS) × sensor location interaction		**	
State (PD ON vs HS)	*	**	**
Sensor location	**	**	**
State (ON/HS) × sensor location interaction		**	

*p < 0.05 (ANOVA); **p < 0.01 (ANOVA).

*PD ON, Parkinson’s disease patients in ON motor status; PD OFF, Parkinson’s disease patients in OFF motor status; HSs, healthy subjects*.

There was a significant main effect of ON/OFF state [*F*(1) = 16.087; *p* = 0.002] and of sensor location [*F*(4) = 33.45; *p* < 0.001] on total power index, with a significant interaction between ON/OFF state and sensor location on this index [*F*(4) = 7.684; *p* < 0.001]. *Post hoc* analysis showed the following sensors location significances, index *p* = 0.003; thumb *p* = 0.001; wrist *p* = 0.005 (Figure 9).

ANOVA analysis showed a significant main effect of OFF/healthy state [*F*(1; 23) = 34.776; *p* < 0.001] and of sensor location [*F*(4; 92) = 84.44; *p* < 0.001] on this index, with a significant interaction between OFF/healthy state and sensor location on this index [*F*(4) = 22.31; *p* < 0.001]. *Post hoc* analysis showed the following sensors location significances, index, wrist, and metacarpus *p* < 0.0001; thumb *p* = 0.0003, and arm *p* = 0.0002 (Figure 9).

ANOVA analysis showed a significant main effect of ON/healthy state [*F*(1; 23) = 23.892; *p* < 0.001] and of sensor location [*F*(4; 92) = 93.198; *p* < 0.001] on this index, with a significant interaction between ON/healthy state and sensor location on total power index [*F*(4) = 13.583; *p* < 0.001]. *Post hoc* analysis showed the following sensors location significances, index, wrist, and metacarpus *p* < 0.0001; thumb *p* = 0.001; arm *p* = 0.0004 (Figure 9).

The total power well correlates with the UPDRS item 25 score assigned. The sensor located on the index finger and thumb showed correlation in both OFF and ON motor condition (index OFF *R*^2^ = 0.35; ON *R*^2^ = 0.34; thumb OFF *R*^2^ = 0.35; ON *R*^2^ = 0.35), while for the wrist and metacarpus there was a correlation only in OFF motor status (wrist OFF *R*^2^ = 0.36; metacarpus OFF *R*^2^ = 0.38).

#### Smoothness

Since smooth and well-coordinated movements are typical features of a healthy and well-developed human motor behavior, we expect that the intake of the medication should be assessed by smoothness values near to zero.

**Figure 10:**
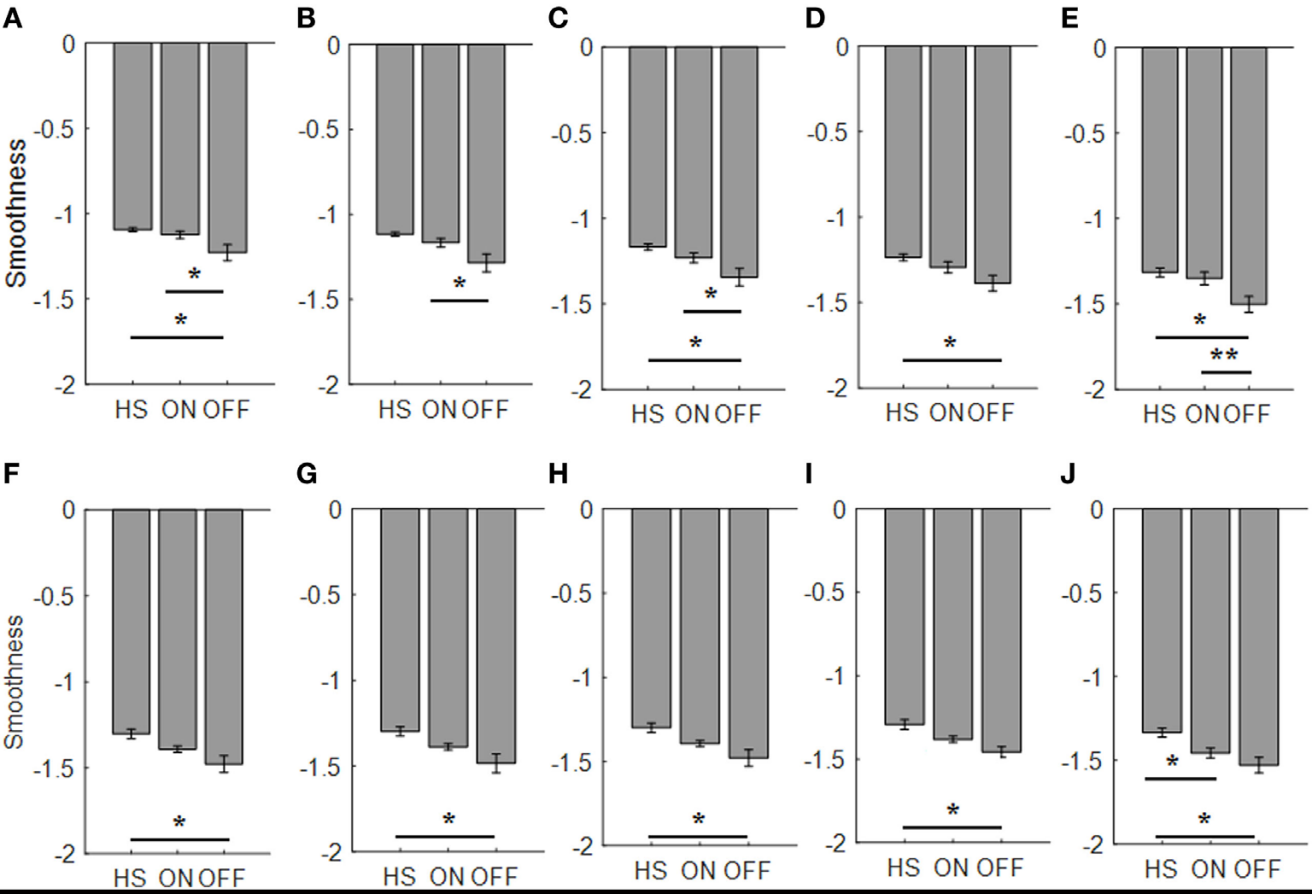
Smoothness index. Each panel shows the averaged values for each group OFF, ON, and healthy subject (HS) for index finger [**(A)**: finger tapping; **(F)**: arm prono-supination], thumb [**(B)**: finger tapping; **(G)**: arm prono-supination], metacarpus [**(C)**: finger tapping; **(H)**: arm prono-supination], wrist [**(D)**: finger tapping; **(I)**: arm prono-supination], arm [**(E)**: finger tapping; **(J)**: arm prono-supination]. Bars denote the SE. *Bonferroni correction *p < 0.01; **p < 0.001.

For the finger-tapping task (Table 4), ANOVA analysis showed a significant main effect of ON/OFF state [*F*(1) = 16.984; *p* = 0.002] and of sensor location [*F*(4) = 157.654; *p* < 0.001] on smoothness index, without an interaction between ON/OFF state and sensor location on this index [*F*(4) = 2.33; *p* = 0.071]. *Post hoc* analysis showed the following sensors location significances, index finger *p* = 0.003; thumb *p* = 0.004; metacarpus *p* = 0.005; arm *p* = 0.0001 (Figure 10).

ANOVA analysis, showed a significant main effect of OFF/healthy state [*F*(1; 23) = 11.427; *p* = 0.003] and of sensor location [*F*(4; 92) = 138.011; *p* < 0.001] on smoothness index, without an interaction between OFF/healthy state and sensor location on this index [*F*(4) = 1.58; *p* = 0.186]. *Post hoc* analysis showed the following sensors location significances, index *p* = 0.003; metacarpus *p* = 0.006; wrist *p* = 0.008; arm *p* = 0.002 (Figure 10).

There was no significant effect of ON/healthy state on smoothness index.

For arm prono-supination task (Table 5), ANOVA analysis showed a significant main effect of ON/OFF state [*F*(1) = 5.025; *p* = 0.047] and of sensor location [*F*(4) = 8.409; *p* < 0.001] on smoothness index, without an interaction between ON/OFF state and sensor location on this index [*F*(4) = 0.607; *p* = 0.659]. *Post hoc* analysis showed no statistically significant differences among sensors (Figure 10).

ANOVA analysis showed a significant main effect of OFF/healthy state [*F*(1; 23) = 12.089; *p* = 0.002] and of sensor location [*F*(4; 92) = 7.315; *p* < 0.001] on this index, without an interaction between OFF/healthy state and sensor location on smoothness index [*F*(4) = 0.514; *p* = 0.726]. *Post hoc* analysis showed the following sensors location significances, index, and metacarpus *p* = 0.005; wrist *p* = 0.001, thumb *p* = 0.008, and arm *p* = 0.002 (Figure 10).

ANOVA analysis showed a significant main effect of ON/healthy state [*F*(1; 23) = 8.271; *p* = 0.009] and of sensor location [*F*(4; 92) = 15.072; *p* < 0.001] on this index, without an interaction between ON/healthy state and sensor location on smoothness index [*F*(4) = 1.354; *p* = 0.256]. *Post hoc* analysis showed the following sensors location significances, arm *p* = 0.005 (Figure 10).

No correlation was found with the arm prono-supination task, between the UPDRS item 25 score and the smoothness index, but there was a good correlation, between UPDRS item 23 score in ON motor condition and the smoothness index for sensors placed on wrist (*R*^2^ = 0.36), thumb (*R*^2^ = 0.47), metacerpus (*R*^2^ = 0.49), and arm (*R*^2^ = 0.43) during the finger-tapping task.

**Figure 11:**
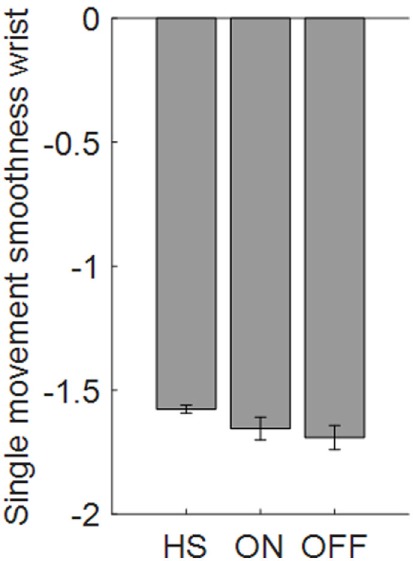
Smoothness index computed from the wrist in the rigidity task. Values are averaged for each group. Bars denote the SE.

Taking into account, the relationship between elbow rigidity and the smoothness index, results showed no discrimination ability between PD OFF condition and ON condition, or PD and HSs (Figure 11). In addition, no correlation between UPDRS item 22 score and smoothness index was found for rigidity.

## Discussion

In the last years, a huge number of studies were published about the quantitative analysis of movement in PD (9, 14–16, 20). The variety of indicators extracted by accelerometer, gyroscope, compass, and other sensors signal in the literature is high. Nevertheless, several questions are still open, since we have not yet reached the stage of a consensus about: which kind of sensor is more suitable for evaluating PD patients, and if it is better to have a single index for each Parkinsonian symptom, or a global index of impairment; where is the best place on the body to wear these sensors, the level of invasiveness acceptable by the patient for at home long-term recording. A recent review (9), focused on published research papers on wearable technologies for PD in the last 10 years, showed that among the 848 analyzed studies only the 6% presents a reliable quantitative assessment system ready for clinical use in the next future. However, a huge number of studies present proof of concepts that could become useful for clinical use in the next years.

An example of an experimental portable device is an instrumented glove used to quantify motor symptoms during deep-brain stimulation surgery (14); authors used only one six-axis IMU placed on the middle finger for tremors and bradykinesia assessment. In this study, five UPDRS motor tasks addressing the upper arm were analyzed (rest tremor, postural tremor, finger-to-nose, repeated hand movements, and rigidity). Experimental results showed that their system is reliable for tremor amplitude determination and movement angles measurement only. Such a similar device was also proposed by Di Pino et al. (21).

A network of uniaxial accelerometers—four located on the upper limbs and four on the lower limbs was proposed by Patel et al. (15). Data were acquired during the execution of UPDRS motor tasks including finger-to-nose, finger tapping, repeated hand movements, heel tapping, sitting, and alternating hand movements. The results of the study, indicated that it is possible to reliably estimate clinical scores on the basis of four features such as root mean square value of accelerometers, data range value of accelerometers, dominant frequency, and the ratio of energy of the dominant frequency component to the total energy. Although differences were observed, several motor tasks performed equally well. This suggests that the proposed parameters capture aspects of the movement patterns that are not specific for a given motor task. This further suggests that the proposed analyses could be extended to activities of daily living.

Heldman et al. (20) used two six-axis motion sensors located on the index finger and thumb, and analyzed only the gyroscope signals. The authors tested PD patients in the OFF and ON motor condition while performing bradykinesia tasks (e.g., finger tapping, hand grasping, and pronation supination). The authors showed a correlation among UPDRS scores and kinematic measures: speed of movements was correlated with log of root mean square of the angular velocity; amplitude of movements was correlated with the root mean square of the excursion angle; and movement rhythm was correlated the coefficient of variation. Their results suggest that motion sensors can objectively measure speed, amplitude, and rhythm and that they are highly correlated with clinician scores.

The state of the art of quantitative assessment tools for PD clearly shows that interactive motor tasks recorded using wearable magneto-inertial devices, allowed to deeply analyze the kinematic and dynamic characteristics of goal-directed movements of upper limb, and to extract quantitative and useful indices for the motor symptoms evaluation.

With the present study, we have searched answer to open questions, which slow the progression to clinical application of the available technologies.

The first question was, where is the best place where to locate sensors. By using a redundant number of upper arm sensors (index finger, thumb, metacarpus, wrist, and arm), our results showed that a distal location of sensors on upper arm (i.e., on index finger) is more sensible to catch the kinematic features of Parkinsonian movements. The following questions were, which index can better differentiate PD patients OFF from ON motor condition and patients in these two conditions from HSs. Our results introduced new indexes that well describe the clinical motor symptoms, and are able to differentiate PD ON/OFF condition and PD vs HSs.

For the first time, we have provided a complete kinematic description of the classic definition of bradykinesia (4) through different quantitative kinematic indexes: the “slowness of voluntary movement” was well described by the total time needed (seconds) to complete a task (the finger-tapping or the arm prono-supination task), and the “progressive reduction in speed and amplitude of repetitive actions,” was well described by a new kinematic index, defined fatigability index. These two kinematic indexes are able to discriminate the ON from the OFF motor condition in PD patient. Moreover, in order to describe bradykinesia, the prono-supination task seems to be the most informative, since with this simple task we can discriminate PD ON vs OFF motor condition (with any sensor location among thumb or wrist), and in addition we can discriminate PD patients in any of these two conditions from HSs. The intrinsic features of prono-supination task, which involves an highest number of muscles, leads to a more versatile task, able to describe the variability of Parkinsonian movement, with sensors placed in different location on upper arm. Conversely, the features of finger-tapping task, lock its utility to the sensor location on the index finger, in this case the results showed a good discrimination ability to distinguish the PD ON vs OFF motor condition.

Overcoming the classic bradykinesia definition, we have described the kinematic of Parkinsonian movement with further two indexes. In order to describe the overall “intensity” of movement, we have extracted the total power that is the power spectrum of the frequency of movement during finger-tapping and prono-supination task. Also for this kinematic index the prono-supination task showed to be more informative compared with other condition, since the total power index can discriminate PD ON vs OFF motor condition and PD patients in any of these conditions from HSs with any sensor location among index finger, thumb, or wrist. Even, the most proximal sensor (arm) is useful to discriminate HS from PD in OFF condition during the prono-supination task. For finger-tapping task, with a sensor placed on the index finger, the total power can discriminate PD ON vs OFF motor condition, and the later from HSs. Therefore, the results show that using prono-supination task, with sensors placed on index finger, thumb, or wrist, total power index is able to perform a complete PD ON/OFF and PD/HS discrimination. The good performance of this index could be explained from its neurophysiological interpretation. In PD, repetitive movement, are supposed to be arhythmic, other than slow, and characterized from a progressive reduction in speed and amplitude. Therefore, the total power index is a perfect index to catch the arhythmicity and the variability of a movement, since an arhythmic movement will be characterized from a more broad and flat PSD graph compared with a rhythmic movement.

The last index that we proposed for the kinematic analysis, the smoothness index, could be interpreted as a bridge parameter, able to describe features that belong to both bradykinesia and rigidity. This kinematic index describes the fluidity of movements, so that it can catch the features of both the bradykinesia, related to the variation of movements rhythm, caused by interruptions or hesitations during task, as well as the cogwheel rigidity, which fragment and decompose passive movement around the joint. For bradykinesia, the smoothness index during prono-supination task is able to discriminate PD in OFF motor condition form HSs, with any sensor location among index finger, thumb, metacarpus, wrist and arm, and PD in ON motor condition form HSs with sensor placed on arm. During finger-tapping task, the smoothness index is able to discriminate PD in OFF from ON motor condition, with any sensor location except the wrist, and PD in OFF from HSs, with any sensor location except the thumb.

Total power was the only index which showed a good correlation with the related UPDRS score, for both task finger-tapping and arm prono-supination, in the last both condition OFF/ON motor status. These versatile features suggest to explore this index, in future studies, as a candidate to monitor PD motor symptoms. Total time, total power, and smoothness showed a good correlation with the UPDRS score for finger-tapping task, therefore the use of these indexes is suggested only for this task.

## Conclusion

The first aim of the present study was identify the most sensible place where to locate sensors to monitor PD motor symptoms. Our results suggest that a distal location of wearable sensors, on index finger or wrist, should be preferred in these kinds of studies in order to better describe the kinematic features of Parkinsonian movements.

In order to differentiate PD OFF from ON motor condition, the best solution seems to be placing a magneto-inertial sensor on index finger during finger-tapping task, so obtaining data from which to extract the kinematic indexes proposed (total time, total power, or smoothness). In addition, this sensor location guarantees a good correlation between the clinical score as expressed by UPDRS scale and the kinematic measure (total time, total power).

In order to differentiate PD patients from HSs, the total power index, computed from data acquired by a sensor placed in any location among index finger, thumb, metacarpus, wrist, and arm during prono-supination task has shown the best accuracy. However, also total time, during the same task, with any sensor location could be a valid alternative to differentiate PD patients from HSs.

In conclusion, combing all results, our study shows that considering all variables (sensors location; motor task performed; kinematic index analyzed), the most versatile, and complete solution, that could answer to both questions (PD OFF vs ON differentiation and PD vs HS differentiation), with highest accuracy, is to place one sensor on index finger, thumb, or wrist, perform a prono-supination task and use the total power as kinematic index. However, keeping in mind the small sample size of the present study, the proposed indexes, are good candidates to be explored in further confirmation studies with larger population.

## Ethics Statement

This study was carried out in accordance with the recommendations of Good Clinical Practice with written informed consent from all subjects. All subjects gave written informed consent in accordance with the Declaration of Helsinki. The protocol was approved by the ethics committees of IRCCS San Raffaele Pisana and Campus Bio-Medico University of Rome.

## Author Contributions

1. Research project: A. Conception, B. Organization, C. Execution; 2. Statistical Analysis: A. Design, B. Execution, C. Review and Critique; 3. Manuscript Preparation: A. Writing of the first draft, B. Review and Critique; LB: 1A, 1B, 1C, 2C, 3A, 3B. SS: 1B, 1C, 2A, 2B, 2C, 3A, 3B. JT: 1B, 1C, 2C, 3B. FT: 1A, 1B, 2C, 3B. MM: 1B, 1C, 2C, 3B. AR: 1C, 2C, 3B. FV: 1A, 1B, 2C, 3B. DF: 1A, 1B, 2A, 2C, 3B. VL: 1B, 2C, 3B. GP: 1A, 1B, 2C, 3B. MT: 1A, 1B, 2C, 3B.

## Conflict of Interest Statement

The authors declare that the research was conducted in the absence of any commercial or financial relationships that could be construed as a potential conflict of interest. The handling editor declared a past co-authorship with one of the authors (VL) and states that the process nevertheless met the standards of a fair and objective review.
